# Numerical Study on Transient State of Inductive Fault Current Limiter Based on Field-Circuit Coupling Method

**DOI:** 10.3390/ma12172805

**Published:** 2019-08-31

**Authors:** Wenrong Li, Jie Sheng, Derong Qiu, Junbo Cheng, Haosheng Ye, Zhiyong Hong

**Affiliations:** 1 School of Electronic Information and Electrical Engineering, Shanghai Jiao Tong University, Shanghai 200240, China; 2 Russian Representative Office of State Grid Corporation of China, Moscow 109807, Russian

**Keywords:** inductive fault current limiter, magnetic flux shielding, multiphysics simulation, transient state, field-circuit coupling method

## Abstract

As the capacity of the power grid continues to expand, high-level fault currents might be caused during a contingency, and the problem of short-circuit current over-limitation is imminent. The high-temperature superconducting (HTS) fault current limiter (FCL) is an effective method to solve this problem. In this paper, a transient numerical model for the process of limiting current in the inductive FCL is proposed. The model is based on the coupling of multiphysics finite element simulation and a circuit model. The voltage source is used as input, which can simulate the macroscopic characteristics in the process of limiting current, such as the voltage and current waveforms, and can also simulate microscopic characteristics, such as temperature, magnetic field, and electrodynamic force distribution. The short-circuit experimental data of an air core inductive superconducting fault current limiter (SFCL) prototype was compared with the simulation results to verify the reliability of the simulation.

## 1. Introduction

With the increasing power load and increased short-circuit capacity, the short-circuit current of the grid will continue to rise and will gradually approach the limits of the breaking capacity, which will greatly affect the stability of the power system and equipment. Among the current-limiting equipment, the superconducting fault current limiter (SFCL) is considered as one of the most effective methods to solve the problem of exceeding short-circuit current. Thus, simulating and conducting experimental research on it is significant [1].

SFCLs can be divided into resistive, inductive, three-phase reactor, saturated iron core, and bridge types. The principle of resistive SFCL is the transition of the superconductor from superconducting to normal state. They are relatively simple in structure, but continuous losses during rated operation is unavoidable. The inductive SFCL omit the current leads, but in general use an iron core, which makes this SFCL relatively heavy and costly. Recently, the inductive SFCL has received more attention [2,3,4,5]. 

In 1996, the Swiss ABB technology company developed a 1.2 MVA/10.5 kV three-phase inductive high-temperature superconducting fault current limiter (HTS FCL), installed it in a hydropower station in Lausanne, and carried out a durability test for one year. It was the first superconducting electrical equipment tested under the actual operating conditions of a power station [6]. The secondary winding is a superconducting magnetic shielding ring made of BSCCO-2212, cooled by liquid nitrogen. In the test, the peak value of short-circuit current was limited to a range from 60 kA to 700 A. The Warsaw Institute of Superconductivity in Poland and the Karlsruhe Institute of Technology in Germany developed an air-core inductive FCL. In 2016 and 2018, 15 kV/140 A [7] and 10 kV/600 A [8] single-phase inductive FCLs were developed. 

The inductive current limiter simulation is divided into a steady-state simulation for rated operation and a transient-state simulation for the current-limiting process. When the SFCL is in rated operation, the temperature variation of superconducting winding can be neglected, because the HTS tapes do not generated large heat quickly. The steady-state simulation can be based on the numerical model using the H formulation [9,10] or the T-A formulation [11,12]. These numerical models usually use the finite element method (FEM) or the finite difference method to solve Maxwell equations (including Gauss’s law for magnetism, Faraday’s law of induction, and Ampere’s law with Maxwell’s addition); the detailed derivation process is shown in the above references. The input of the simulation model is current, and the output is the current density distribution, magnetic field distribution, and AC loss in the SFCL. When the SFCL is in the current-limiting process, the temperature of the superconducting winding rises significantly, affected by the short-circuit current. Since impedance of the superconducting winding is affected by current, magnetic field, and temperature simultaneously, multiple factors are combined, so a transient simulation of the inductive FCL is more difficult.

In [13], resistance connected in parallel with a switch was used to simulate the resistive component of the superconducting winding, and the secondary induced current was calculated by the finite element simulation based on an electromagnetic model. The advantage of the model is that it provided fast computation speed and could simulate the macroscopic working characteristics of the current limiter, including voltage and current curves. However, since the model, lumped parameter model, equated the resistive component of tape to the parallel connection of the resistor and the switch, only the equivalent parameter value can be obtained. It was impossible to simulate microscopic working characteristics, such as the distribution of current and temperature. In [14,15,16], the magnetic field shielding characteristics of a single inductive current limiter unit in air or iron core were measured at different currents, and then equivalent inductance of the entire current limiter was estimated from the angle of the alternating magnetic flux and the winding hinge. The calculation result was used in the circuit model calculation. Since the model combined the measured values, the calculation results were more accurate. However, the measured value of a single current-limiting unit cannot fully represent the electromagnetic environment of the current limiter. At the same time, since the current-limiter impedance was calculated by the overall equivalent method, it was impossible to simulate the microscopic properties of the tape.

In this paper, a transient numerical model for the process of limiting current in the inductive FCL is proposed. This numerical model including three main models: Circuit model, electromagnetic model, and heat transfer model. Then, an optimization method to accelerate convergence velocity of iteration is proposed. Based on the model, several typical examples are discussed, including the following parameters: The current and voltage, magnetic field and electrodynamic force distribution, and temperature distribution. A SFCL prototype is fabricated, and a short-circuit test of the SFCL is compared with the simulation results to verify the reliability of the simulation.

## 2. Methodology of Numerical Model

### 2.1. Model Overview

When a short-circuit fault occurs in the power grid, the short-circuit current will far exceed the critical current in the current limiter. Meanwhile, part of the current will flow through the metal layer, and a large amount of joule heat will cause a significant temperature rise in the winding. Therefore, to calculate the temperature change inside the winding, the temperature field in a simulation model is needed, which is combined with the electromagnetic model to calculate the current-limiter impedance. In addition, in actual situations, the voltage value of the transformer is known instead of the short-circuit current, so the circuit model is used to calculate the instantaneous short-circuit current based on the power voltage and current-limiter impedance. Since the calculation results of multiple physical fields involved in the whole model are interdependent (such as current and current-limiter impedance), multilayer iterative calculations are needed to converge the model and simulate the operating characteristics of the actual current limiter [17,18]. 

The sophisticated commercial FEM software can solve the complex problems quickly and accurately. The ability of interactive post-processing and visualization make the analyzing of the results easier. The FEM software being used in this paper is COMSOL multiphysics 3.0, and both electromagnetic model and heat transfer model are based on the software. The circuit model is calculated by MATLAB.

**Figure 1:**
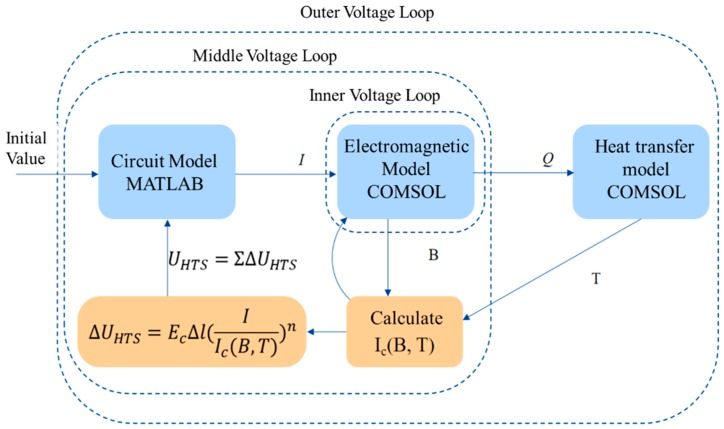
Schematic diagram of inductive superconducting fault current limiter (SFCL) transient-state simulation model.

The overall calculation structure of the model is shown in Figure 1.

### 2.2. Circuit Model

**Figure 2:**
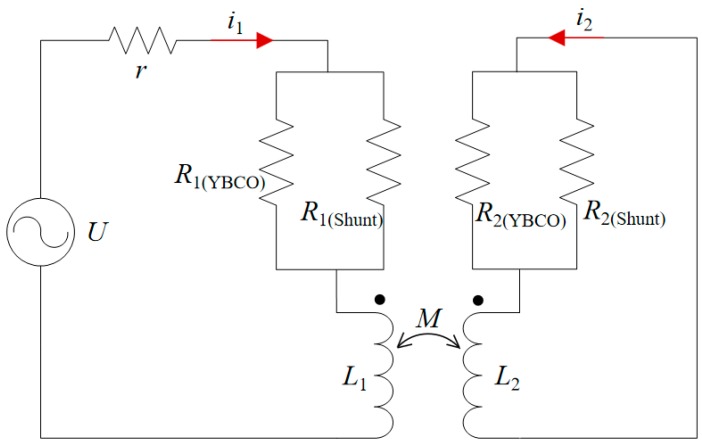
Schematic diagram of circuit model.

The SFCL model can be equivalent to a transformer with a secondary short circuit. The circuit model of the SFCL is shown in Figure 2.

In Figure 2, *U* is the power voltage, *r* is the line resistance, *i*_1_ and *i*_2_ are the primary and secondary currents, respectively, *R*_1(*YBCO*)_ and *R*_1(*Shunt*)_ are the equivalent resistance of the HTS layer and metal layer in primary winding, respectively, *R*_2(*YBCO*)_ and *R*_2(*Shunt*)_ are the same as above in the secondary winding, *L*_1_ and *L*_2_ are self-inductance of the primary and secondary winding, respectively, and *M* is mutual inductance.

In this model, the calculation of resistance is equivalent to the calculation of the resistance voltage drop. The resistance of the primary and secondary windings is in parallel with the resistance of the HTS layer and the metal layer.

For the HTS layer, the resistive voltage drop is related to the temperature, amplitude, and direction of the magnetic field. Because of the nonuniform magnetic field and different positions of the primary and secondary windings, it is necessary to separately calculate the resistive voltage drop of each turn, and then accumulate the entire winding to obtain the total resistive voltage drop, as shown in Equation (1):

(1) $$U={\sum_{N}E_{c}\Delta l\left(\frac{I_{YBCO}}{I_{c}(B,T)}\right)}^{n}$$

where *N* is the total number of turns, *Δl* is the length of each turn, *E_c_* is the critical electrical field, usually taking a constant value 1 × 10^−6^ V/m, n value is 29 for the tape used in this study. *I_YBCO_* is the current of the HTS layer, and *I_c_*(*B*, *T*) is the critical current under magnetic field *B* and temperature *T*, as shown in Equation (2) [19]:

(2) $$I_{c}\left(B,T\right)=\frac{I_{c}\left(T\right)}{{\left[1+\sqrt{{\left(kB_{∥}\right)}^{2}+B_{⊥}^{2}}/B_{c}\right]}^{\alpha }}$$

In the formula, $B_{∥}$ is the components of the magnetic field parallel to the superconducting surface, $B_{⊥}$ is the components of the magnetic field vertical to the superconducting surface, *k*, *α*, *B_c_* are physical parameter of superconducting tape, which represent the degradation of critical under different background magnetic field, and the value of *k* is between 0 and 1. In this paper, according to the test results of the critical current magnetic field angle-dependency of the YBa_2_Cu_3_O_7-x_ (YBCO) tape, *k* = 0.3672, *α* = 0.6267, and *B_c_* = 0.05249 T.

For the metal layer, resistivity is only related to temperature, but since it is composed of various metals, the resistivity of each metal varies with temperature. The resistivity of superconducting tape as a function of temperature is measured when it is above the critical temperature. Below the critical temperature, a linear cubic spline fit is made at 92 K based on the measured data. 

In summary, the overall circuit model formula can be obtained, and discrete iterations are used in the actual calculation, as shown in Equation (3):

(3) $$\{\begin{array}{c} U_{\left(m\right)}=i_{1\left(m\right)}r+\sum_{N_{1}}E_{c}\Delta l{\left(\frac{i_{1\left(YBCO\right)\left(m\right)}}{I_{c}\left(B,T\right)}\right)}^{n}+L_{1}\frac{i_{1\left(m\right)}-i_{1\left(m-1\right)}}{\Delta t}-M\frac{i_{2\left(m\right)}-i_{2\left(m-1\right)}}{\Delta t}\\ 0=\sum_{N_{2}}E_{c}\Delta l{\left(\frac{i_{2\left(YBCO\right)\left(m\right)}}{I_{c}\left(B,T\right)}\right)}^{n}+L_{2}\frac{i_{2\left(m\right)}-i_{2\left(m-1\right)}}{\Delta t}-M\frac{i_{1\left(m\right)}-i_{1\left(m-1\right)}}{\Delta t} \end{array}$$

In the formula, *i*_1(*YBCO*)(*m*)_ is taken as an example, the number 1 represents the primary side, _(*m*)_ represents the *m*th step iteration, and _(*YBCO*)_ represents the HTS layer, so *i*_1(*YBCO*)(_*_m_*_)_ represents the current of the HTS layer in the primary winding at the *m*th iteration; *i*_2(*m−*1)_ represents the total current in the secondary winding at the *(m−*1*)*th iteration; *N*_1_ and *N*_2_ is the total number of primary winding and secondary winding, respectively, and Δ*t* is the size of the iterative step (1 × 10^−5^ s). 

### 2.3. Electromagnetic Model

The electromagnetic model is based on the COMSOL AC/DC module, and it is based on Maxwell equations, as shown in Equation (4):

(4) $$\{\begin{array}{c} \nabla \cdot B=0\\ J=\nabla \times H\\ \nabla \times E=-\frac{\partial B}{\partial t} \end{array}$$

where *B* is the magnetic flux density, *J* is the current density, *H* is the magnetic field strength, *E* is the electric field strength, and *t* represent time.

**Figure 3:**
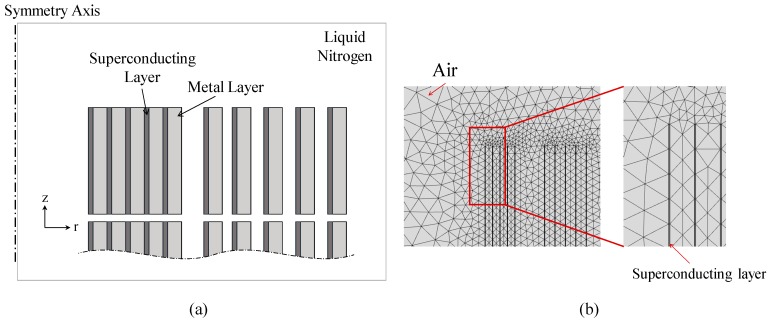
Schematic diagram of electromagnetic model. (**a**) Structure; (**b**) mesh.

Figure 3 shows a schematic diagram of the electromagnetic model, which is a two-dimensional axisymmetric graph. Figure 3b is a diagram with mesh generation in simulation, the thickness of the superconducting layer is magnified 10 times to accelerate the simulation. About mesh generation in superconducting layer, 20 grids in z direction and 1 grid in r direction are generated. 

In order to simplify the model and speed up the calculation, $B_{∥}$ (Equation (2)) is the average of parallel magnetic field in one turn, and $B_{⊥}$ (Equation (2)) is the average of vertical magnetic field in one turn. Differences in current density at different points are ignored for the same turn. The electromagnetic model is used to calculate the *I_YBCO_* and *I_shunt_* when the total current is determined.

The specifications of the SFCL model are shown in Table 1.

### 2.4. Heat Transfer Model

**Figure 4:**
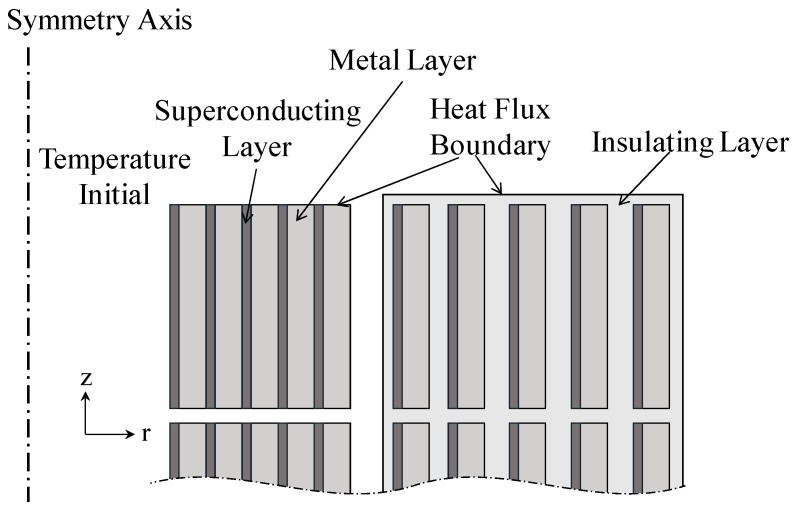
Schematic diagram of heat transfer model.

The heat transfer model is based on the COMSOL heat transfer in solids module. As shown in Figure 4, a heat flux is applied to the outer boundary to simulate the process of boiling liquid nitrogen heat transfer, so that the simulated liquid nitrogen region can be directly deleted.

The heat transfer model is divided into two parts. The first part is solid heat transfer, including heat transfer inside the superconducting tape, between tapes, and between tape and insulating material. The basic equation is as follows [20]:

(5) $$\rho C_{p}\left(T\right)\frac{\partial T}{\partial t}=\nabla \cdot \left(\lambda \left(T\right)\nabla T\right)+Q$$

where *ρ* is density, *T* is temperature, *C_p_*(*T*) is specific heat capacity, *λ*(*T*) is thermal conductivity, *t* represent time, and *Q* is the heat source in this model, which is the density loss *E·J* of the winding.

In the model, the simulation space is divided into three parts: Superconducting, metal, and insulating layers. The metal layer is composed of multiple layers of different materials, and the specific heat capacity *C_p_*(*T*) and thermal conductivity *λ*(*T*) as functions of the temperature of each layer are different. To simplify the model, uniform thermal parameters are needed. *C_p_*(*T*) and *λ*(*T*) of each layer are equivalently calculated, as shown in Equations (6) and (7):

(6) $$C_{eq}=\frac{\sum_{N}C_{n}\left(T\right)\rho_{n}t_{n}}{\sum_{N}\rho_{n}t_{n}}$$

(7) $$\lambda_{eq}=\frac{\sum_{N}\lambda_{n}\left(T\right)t_{n}}{\sum_{N}t_{n}}$$

where, respectively, *C_eq_* and *λ_eq_* are equivalent specific heat capacity and thermal conductivity and *C_n_* and *λ_n_* are specific heat capacity and thermal conductivity of each metal layer, and *ρ_n_* is the density and *t_n_* is the thickness of each metal layer. *N* represents the types of mental materials.

The second part of the heat transfer model is the heat exchange between the outer boundary of the winding and the liquid nitrogen, which is dominated by thermal convection. However, since the increased temperature of the winding causes the liquid nitrogen to boil, the heat transfer model is complicated. In order to simplify the calculation, a heat flux is applied to the outer boundary to simulate the process of boiling liquid nitrogen heat transfer, so that the simulated liquid nitrogen region can be directly deleted. The basic equation is as follows:

(8) $$Q=h\left(T_{s}-T_{0}\right)\cdot A\cdot \left(T_{s}-T_{0}\right)$$

where *T_s_* is the temperature of the heat flux boundary, *A* is the boundary cross-sectional area, *T*_0_ is the liquid nitrogen temperature (77 K), and *h (T_s_* − *T*_0_*)* is the effective nonlinear steady-state convection coefficient (W/(K∙m^2^)). The value of *h (T_s_* − *T_0_)* is liquid nitrogen boiling heat flux curve in the simulation, which is cited in [21]. 

### 2.5. Model Convergence and Optimization

#### 2.5.1. Middle Voltage Loop Optimization

As shown in Figure 1, the iterative calculation is divided into three layers: Inner, middle, and outer. The middle voltage loop is used to iterate in the circuit model (calculating the primary and secondary currents) and the electromagnetic model (calculating the resistive voltage drop of the superconducting winding) under a specified supply voltage. The intermediate variable is the superconducting winding resistive voltage drop *U_sc_p_*, *U_sc_s_*, and the updating method is shown as Equation (9):

(9) $$\{\begin{array}{c} U_{sc\_p\left(k\right)}=U_{sc\_p\left(k-1\right)}-\theta \Delta U_{sc\_p\left(k\right)}\\ U_{sc\_s\left(k\right)}=U_{sc\_s\left(k-1\right)}-\theta \Delta U_{sc\_s\left(k\right)} \end{array}$$

where *U_sc_p_*_(*k*)_ represents the primary resistive voltage drop at the *k*th iteration and *U_sc_s_*_(*k*)_ is the secondary voltage drop, where *k* refers to the number of iterations in the middle voltage loop; Δ*U_sc_p_*_(*k*)_ is the difference between *U_sc_p_*_(*k*)_ and *U_sc_p_*_(*k−*1)_; and *θ* is the learning rate. In order to accelerate convergence in the early stage, a method of exponential decay plus constant is used to calculate *θ*, such as Equation (10):

(10) $$\theta \left(k\right)=\beta e^{-\frac{k}{\tau }}+\xi$$

where *β* is the exponential decay coefficient, and the value is large (0.2), which ensures that the model can be quickly converged at the beginning; *τ* controls the decay rate; and *ξ* is the constant learning rate, which ensures that the learning rate of the model does not decay to a too small value after multiple iterations and cannot update. In the simulation, *β* = 0.2, *τ* = 30, and *ξ* = 0.01.

#### 2.5.2. Inner Voltage Loop Optimization

The inner voltage loop is used to calculate the current distribution in the superconducting and metal layers under a specified total winding current. In the actual calculation, due to the characteristics of the HTS, its current will enter a large nonlinear region when it reaches the critical current, as shown in Equation (1). At this time, if only *U_sc_p_* and *U_sc_s_* are used as the convergence conditions, the model will oscillate easily and cannot converge. 

To solve this problem, when the total winding current is close to or greater than the critical current, *U_metal_p_* and *U_metal_s_* are used as convergence conditions. The superconducting layer has nonnegligible resistance at this time, a part of current flow through the metal layer, which results in a nonnegligible voltage drop in the metal layer, and this voltage drop can be directly calculated. Because the resistance of the metal layer is constant at the same temperature, there is no oscillation. 

**Figure 5:**
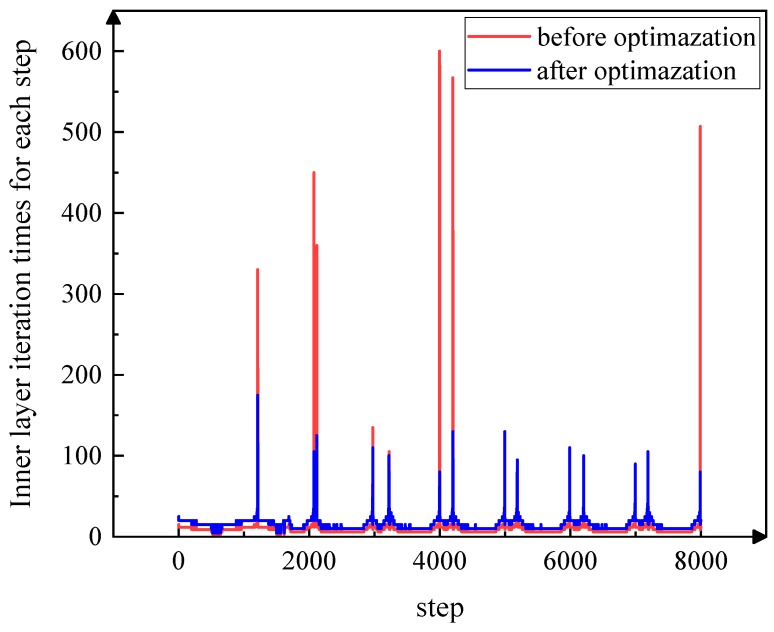
Number of iterations at each step before optimization and after optimization.

Figure 5 shows the number of iterations per step in two cycles (40 ms) before and after optimization. The ordinate represents the iterations of the inner current loop when the outer voltage loop is iterated once; the abscissa is the iterations of the outer voltage loop. Figure 5 shows that the number of iterations at some special points can be greatly reduced after optimization.

## 3. Results and Discussion 

### 3.1. Typical Examples

#### 3.1.1. Current and Voltage

The primary and secondary current curves at different supply voltages were obtained and used to analyze the macroscopic operating characteristics of the SFCL in the simulation. The operating conditions under two typical voltages (voltage amplitudes of 0.5 V, 30 V) are listed, including primary current, primary superconducting layer current, secondary current, secondary superconducting layer current, and voltage.

**Figure 6:**
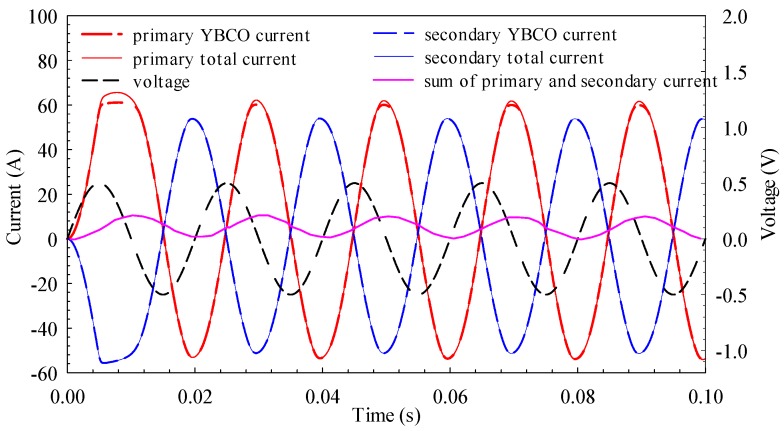
Simulation results of voltage and current curves at voltage magnitude of 0.5 V.

Figure 6 shows the simulation results of the voltage and current curves at a voltage magnitude of 0.5 V. The current flows from the superconducting layer before the primary current reaches 60 A for the first time; after the current exceeds 60 A, the shunting phenomenon begins to appear. It shows that the superconducting layer begins to show obvious resistance because its current exceeds the critical current. However, compared with the metal layer resistance, this resistance is still small, so only a few amperes of current flow through the metal layer.

When the waveform is stable, the primary current amplitude is 53.9 A, the secondary current amplitude is 51.2 A, and the phase difference between the two is 174.6°. In order to explain the shielding effect of the secondary winding on the magnetic flux generated by the primary winding, the sum of the primary and secondary currents is defined as “net current,” which generates magnetic flux (the ratio of primary to secondary is 1:1). The purple curve in Figure 6 is net current; the amplitude is 5.1 A and bias is 6.0 A, which is 9.5% of the primary current. The secondary winding shields most of the magnetic flux generated by the primary winding. Only 9.5% of the primary current builds an alternating magnetic field, so the overall leakage inductance is close to zero. The primary and secondary currents flow through the HTS layer, so the overall impedance of the SFCL is close to zero. The phase difference between the primary current and the voltage waveform is 84.6°. The leakage inductance of the primary and secondary windings makes the SFCL inductive.

**Figure 7:**
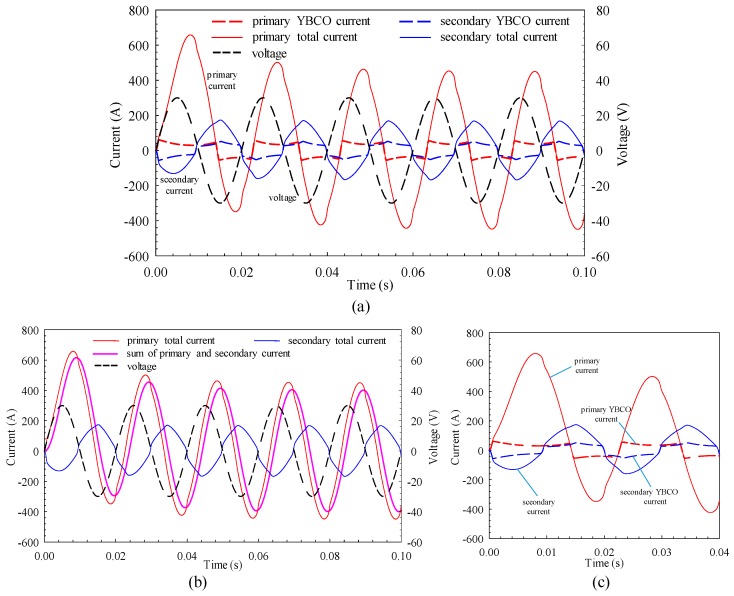
Simulation results of voltage and current curves at voltage magnitude of 30 V: (**a**) Overall curves, (**b**) net current that generates magnetic flux, (**c**) details of curves at first two cycles.

Figure 7 shows the simulation results of the voltage and current curves at a voltage magnitude of 30 V. The first peak of the primary current is 652.7 A, and the current wave becomes steady state in the fifth cycle with the peak value of 427.4 A. After the current of the secondary winding becomes stable, the peak value is 127.1 A, and the amplitude of net current generating the alternating magnetic field is 399.5 A, which is 93.5% of the primary current. It indicates that most of the primary current is used to construct the alternating magnetic field, and the secondary current can only shield a fraction of the magnetic flux generated by the primary current. The SFCL has large inductance; meanwhile, the resistive component of the winding is also increased due to the large primary current. The phase difference between the primary current and the voltage is 57.6° in steady state.

At this moment, due to the large primary and secondary current, a considerable amount of joule heat is generated, and the influence of the increased winding temperature on the HTS strip cannot be ignored. As shown in Figure 7a, the heat accumulation results in decreased saturation currents in the primary and secondary superconducting layers, because the critical current decreases as the winding temperature rises, and the equivalent impedance increases. The temperature rise curve and temperature distribution of the winding will be analyzed in detail later.

#### 3.1.2. Magnetic Field and Electrodynamic Force

**Figure 8:**
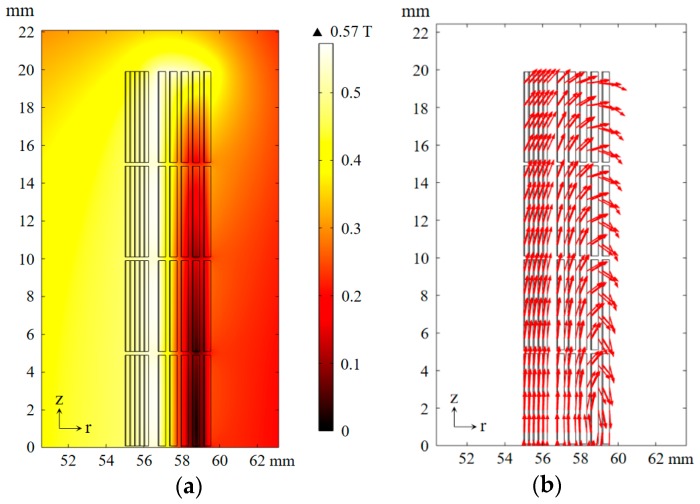
Magnetic field distribution at first current peak of primary winding under voltage magnitude of 30 V: (**a**) Flux density norm value distribution, (**b**) magnetic field direction diagram.

By analyzing the distribution of the magnetic field, the distribution of critical current can be derived, then the distribution of power density can be inferred. In addition, the maximum electrodynamic force acting on the limiter depends on the first peak of primary current under short-circuit conditions. Figure 8 shows the magnetic field distribution at the first current peak of the primary winding under a voltage magnitude of 30 V. 

In the area between primary and secondary windings, the entire secondary winding, and the inside and ends of the primary winding, the magnetic field is largest. Among them, the secondary winding and inside and ends of the primary winding are mainly influenced by the magnetic field parallel to the tape. According to Equation (2), this magnetic field has little effect on the critical current, which results in a low power loss density; the ends of the primary winding are mainly affected by the magnetic field vertical to the tape, and this results in low critical current density and high power loss density. To improve the recovery speed after cutting off the short fault, the above problem should be taken into consideration in the design of a cooling structure. If necessary, tapes with higher critical current and better cooling structure can be used in the ends of the primary winding. 

**Figure 9:**
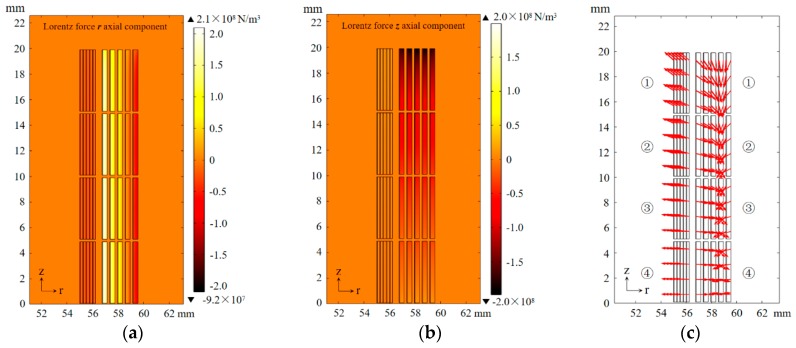
Electrodynamic force distribution at the first current peak of primary winding under voltage magnitude of 30 V: (**a**) R-axial component, (**b**) z-axial component, (**c**) electrodynamic force direction.

Figure 9 shows the electrodynamic force distribution at the first current peak of the primary winding under a voltage magnitude of 30 V. The electrodynamic force affecting the secondary winding is small and the direction is negative; in the primary winding, the inner side is influenced by positive force and the outer side, and the winding is squeezed in the r-axis direction. For electrodynamic force in the z-axis direction, the secondary winding has a small influence and the direction is positive; in the primary winding, the end is greatly affected by a negative force and the middle is influenced by a positive force, and the winding is squeezed in the z-axis direction.

As shown in Figure 9c, different parts of the winding are influenced by electrodynamic force in different directions. Electrodynamic force is a macro performance of the Lorentz force; the magnetic field of the conductor is generated by its own current, so the direction of electrodynamic force does not change with the direction of the current.

To ensure that the SFCL will not be destroyed in the structure under short-circuit current, the electrodynamic force affecting different parts of the winding should be considered in the design. The superconducting coils in the primary and secondary windings of the upper half of the SFCL are numbered and calculated by the electrodynamic force they affect; the numbers are shown in Figure 9c and the calculation results are shown in Table 2.

The force acting on the secondary winding is not large, and the external shedding force in the z-axis should be considered in the design; the force acting on the primary winding is large, more attention should be paid to the outward force, and it is necessary to enhance the structure because of the large force.

#### 3.1.3. Temperature

**Figure 10:**
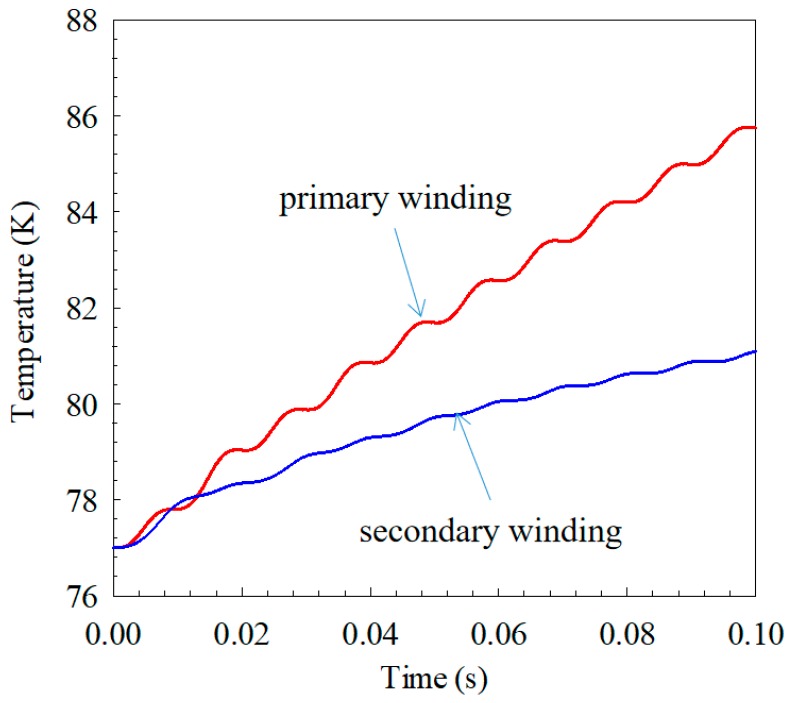
Rising temperature of primary and secondary windings under voltage magnitude of 30 V.

When the short-circuit current is large, a large amount of joule heat generated by winding will result in a significant temperature rise, affecting the electromagnetic properties of the tape. Taking the simulation result voltage magnitude of 30 V as an example, the rising temperature curve of primary and secondary windings is shown in Figure 10. Since the rising temperatures of different turns are different in the same winding, the averages of temperature rise of the primary and secondary sides are used. As shown in Figure 10, the average temperature of the primary winding increases from the initial 77 K to 85.8 K, and that of the secondary winding increases from 77 K to 81.1 K. Figure 6 shows that the primary current is greater than the secondary current, so the primary temperature rise should be greater than the secondary, but because the primary side resistivity is less than the secondary side, the combined action causes a minor difference in temperature rise.

**Figure 11:**
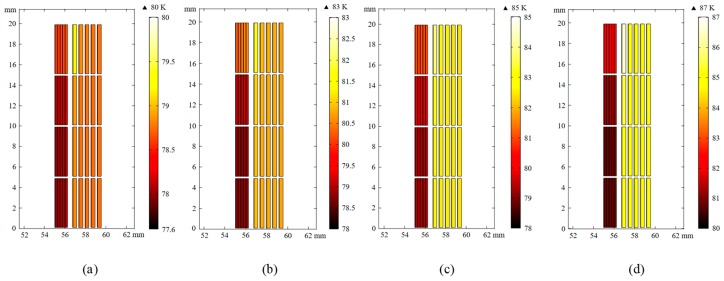
Temperature distribution of primary and secondary windings at (**a**) 0.02 s, (**b**) 0.04 s, (**c**) 0.06 s, and (**d**) 0.08 s.

In order to further analyze the internal operation of the SFCL, Figure 11 shows the temperature distribution of the primary and secondary windings at 0.02 s, 0.04 s, 0.06 s, and 0.08 s (only the upper half of the winding is drawn because it is symmetric).

As shown in Figure 11, the portion with the greatest temperature rise is at the end of the primary innermost winding. Since it is affected by the largest magnetic field (especially the magnetic field perpendicular to the tape), the critical current there is minimized. A large amount of joule heat causes the greatest temperature rise. In the space between the primary and secondary windings, due to the opposite direction of the current, the generated magnetic fields are superimposed and enlarged, but the magnetic field direction is parallel to the strip, so the field has little effect on the critical current. It can be seen that the temperature rises more inside the primary winding than outside. Similarly, in the secondary winding, the temperature rises the most at the end. Since the structure of all-metal and no-insulation layers makes it possible to exchange heat with liquid nitrogen directly, the heat conduction speed is fast. So overall, the temperature rises significantly less in the secondary winding than in the primary winding.

As shown in Figure 11, compared with the secondary winding, the temperature of the primary winding rises more greatly in the current-limiting state of the HTS FCL. In order to speed up the recovery time after the fault current is cut off, the heat dissipation problem should be considered in the actual design and manufacture of the primary winding structure. While ensuring support strength, the contact area between the winding and the liquid nitrogen is increased as much as possible.

### 3.2. Short-Circuit Test

In order to simulate the fault conditions in an actual grid, a SFCL prototype with the same simulation structure is fabricated and connected to the short-circuit test platform. The primary winding is an insulation superconducting winding with insulating material of KAPTON, and the secondary winding is a no-insulation superconducting winding. In this paper, superconducting winding is wound with second-generation high temperature superconducting material—YBCO tapes with stainless-steel package, which are produced by Shanghai Superconductor. 

**Figure 12:**
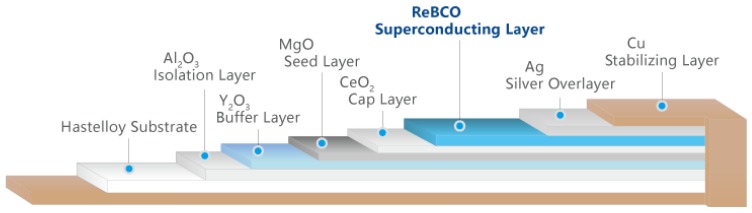
Structure diagram of YBCO tape.

HTS coated conductor (CC) tapes were used in this paper is 4.8 mm wide and critical current is 40 A (77 K, self-field). Superconducting tapes are with a 1.5 μm thick YBCO layer, a 50 μm thick Hastelloy substrate, a 2 μm thick silver cap layer, and a 10 μm + 10 μm thick copper stabilization layer. The structure diagram of YBCO tape is shown in Figure 12. Then, the outermost is packaged in 75 m thick stainless steel.

**Figure 13:**
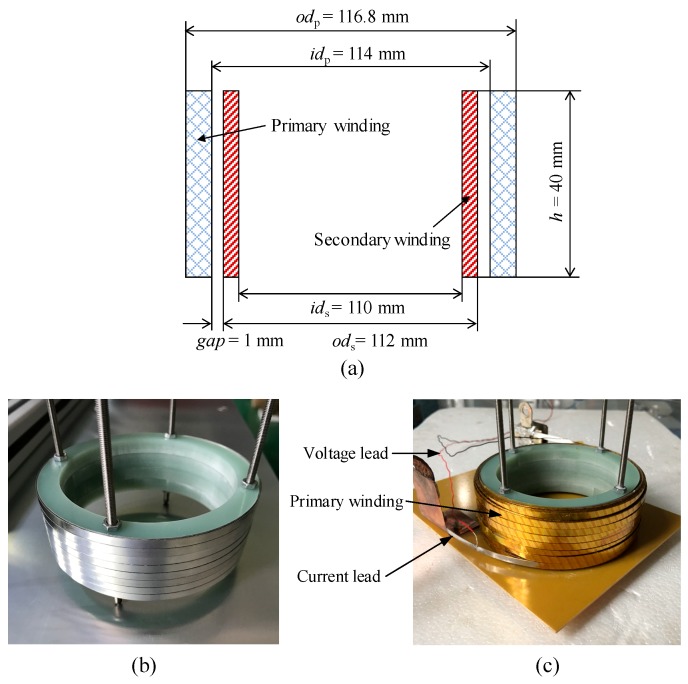
Superconducting inductive SFCL prototype: (**a**) Schematic diagram, (**b**) secondary winding, (**c**) SFCL prototype.

The primary winding and secondary winding consist of four double-pancake coils, respectively. A pancake coil is a coil with a flat spiral form, and a double-pancake coil is two pancake coils made by a superconducting tape. Double-pancake coils are connected in series by soldering, and resistance is 1.5 μΩ. Specifications of the SFCL prototype is shown in Table 1, same with the SFCL model, and the inductive SFCL prototype is shown in Figure 13.

Critical current of primary winding and secondary winding is 28 A and 30 A (77 K), respectively. Self-inductance of primary winding and secondary winding is 0.237 mH and 0.206 mH. The mutual inductance between the primary and the secondary is 0.196 mH, and the coupling coefficient is 0.887. 

**Figure 14:**
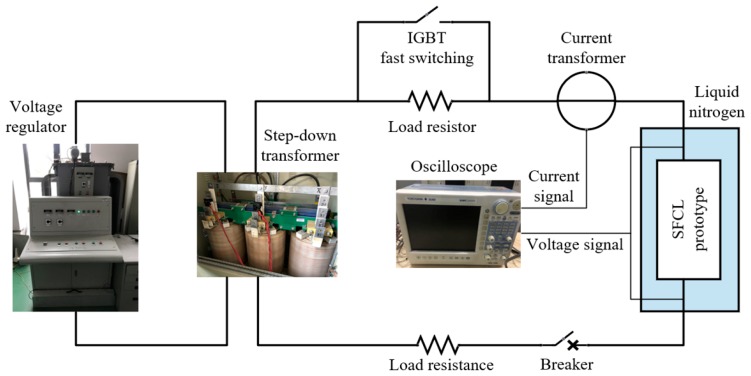
Short-circuit test platform.

The short-circuit test platform is shown in Figure 14; voltage source consists of voltage regulator (input: 400 V, output: 0~400 V) and step-down transformer (ratio is 20:1 or 10:1). Different voltage can be obtained by changing output voltage of voltage regulator. A load resistor connected in parallel with IGBT fast switching is in series with the SFCL prototype, and the IGBT controlled by the host computer is used to generate a short-circuit voltage for a specified duration.

The step-down transformer is used as a voltage source and the turn-off time is controlled to provide a short-circuit current with a fixed time for the SFCL. In this experiment, the short-circuit time is 5 cycles of 100 ms. 

**Figure 15:**
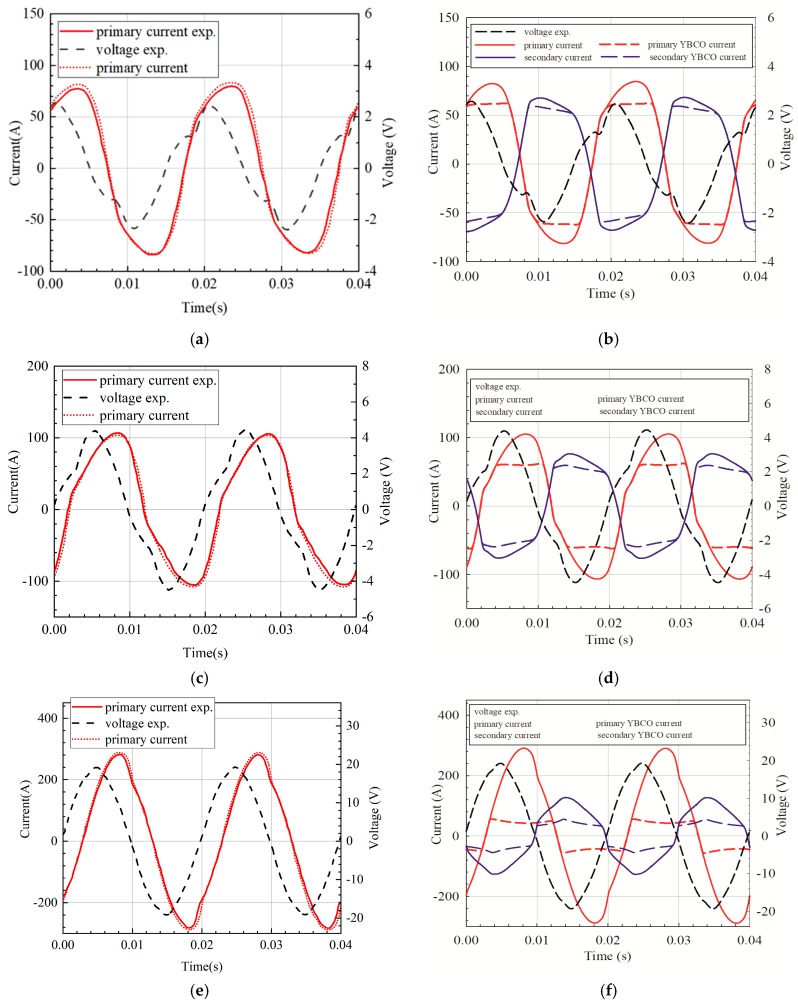
Comparison of experimental (left) and simulation (right) results of voltage and current curves. (**a**) Comparison of primary current curve between experimental and simulation under voltage magnitude of 2.6 V, (**b**) overall simulation current under voltage magnitude of 2.6 V, (**c**) comparison of primary current curve between experimental and simulation under voltage magnitude of 4.5 V, (**d**) overall simulation current under voltage magnitude of 4.5 V, (**e**) comparison of primary current curve between experimental and simulation under voltage magnitude of 19.5 V, and (**f**) overall simulation current under voltage magnitude of 19.5 V.

Considering the difficulty of measuring and simulating the internal impedance of voltage regulator, the experimental voltage value is directly input into the transient model for calculation. Figure 15 shows the comparison of the experimental waveform (left) and the simulated waveform (right) at different voltages (waveforms in two cycles after the short-circuit current becomes steady state). 

As shown in Figure 15, the simulated current waveform is basically consistent with the experimental waveform, which proves that the simulation can well simulate the operating characteristics of the air core current limiter under short-circuit current, which lays a foundation for future SFCL design.

The error between the experiment and the simulation may be caused by the measurement error of the strip electrical and thermal parameters, or by nonuniform electrical and thermal parameters of the strip in the longitudinal direction. The primary current waveform is related to the secondary current waveform, especially when the secondary current begins to shunt in the HTS and metal layers, the secondary side impedance increases, and the instantaneous current value begins to decrease compared with the standard sine wave. At the same time, the primary current is distorted, and the overall distortion is less than the secondary winding.

## 4. Conclusions

In this paper, a numerical study of transient states for the process of limiting current in inductive FCLs is proposed. The model is based on the coupling of multiphysics finite element simulation and a circuit model. By optimization, the number of iterations is reduced, and operational speed is greatly increased. The overall model calculation process, submodel setting, and analysis of the macroscopic characteristics (voltage, current) and microscopic characteristics (distribution of magnetic field, electrodynamic force, and temperature) of the SFCL under the current-limiting state are introduced. In SFCL design, more attention should be paid to the large outward force acting on the primary winding, and it is necessary to enhance the structure; the heat dissipation problem should be considered, while ensuring that the support strength and the contact area between the winding and the liquid nitrogen are increased as much as possible. The short-circuit experimental data of an air core inductive superconducting current limiter (SFCL) prototype was compared with the simulation results to verify the reliability of the simulation and provides a theoretical basis for the future design and manufacture of SFCL. Further, inductive SFCL applied in a short test of a large capability transformer under the grid operating condition can be designed. In this paper, some parameters in electromagnetic model and heat transfer model can be further refined and the simulation accuracy is improved. Similarly, better optimization method can be used to accelerate convergence.

## Figures and Tables

**Table 1:** Specifications of SFCL model.

Item	Specifications	Value
YBCO tape	Tape width	4.8 mm
	Thickness without insulation	0.25 mm
	Ic/n-value (@77 K, self-field)	40 A/29
	Critical temperature	92 K
Primary winding (insulation)	Number of turns	40
	Inner/outer diameter	114 mm/116.8 mm
	Turn-to-turn gap	0.35 mm
Secondary winding (no-insulation)	Number of turns	40
	Inner/outer diameter	110 mm/112 mm
	Turn-to-turn gap	0.25 mm

**Table 2:** Stress of pancake winding under first peak current of primary winding.

	Primary Winding	Secondary Winding
No.	Electrodynamic force r-axial component (N/m)	
1	438.3	−116.6
2	493.4	−138.6
3	517.7	−144.9
4	528.4	−147.1
No.	Electrodynamic force z-axial component (N/m)	
1	−1023.4	75.9
2	−535.8	45.8
3	−283.8	24.7
4	−89.9	7.9
